# Specific PKG inhibitors: do they really exist?

**DOI:** 10.1186/1471-2210-11-S1-P27

**Published:** 2011-08-01

**Authors:** Stepan Gambaryan, Elke Butt, Joerg Geiger, Suzanne M Lohmann, Ulrich Walter

**Affiliations:** 1 Institute of Clinical Biochemistry and Pathobiochemistry, University of Wuerzburg, 97078 Wuerzburg, Germany

## Background

Cellular cGMP effects can be mediated by a number of effectors including cGMP-dependent protein kinases (PKGs), cGMP-stimulated phosphodiesterase (PDE2), cGMP-inhibited phosphodiesterase (PDE3), and cGMP-gated channels. Pharmacological inhibitors of PKG are often used to discriminate between these diverse cGMP effects. Currently used PKG inhibitors can be divided into three classes: cyclic nucleotide binding site inhibitors like Rp-phosphorothioate analogs, ATP binding site inhibitors like KT5823, and substrate binding site inhibitors like the recently described DT-oligopeptides. However, several studies have observed no PKG inhibition by KT5823 in intact cells [1] or by Rp-cGMPS analogs in smooth muscle cells [2], as well as unspecific (PKG-independent) effects of Rp-cGMPS analogs in platelets [3].

## Results

We tested the inhibitory effects of (D)-DT2 and DT3 on PKG and its effects on intact cells. Our data show that (D)-DT2 selectively inhibited 2 nM purified PKG Iα and Iβ with an IC_50_ of 8 nM, and that up to 1 µM (D)-DT2 did not inhibit PKG II or PKA. In broken platelet cell experiments, PKG activity was inhibited by (D)-DT2 starting at 5 µM, with complete inhibition at 20 µM, but we also observed inhibition of PKA activity at these concentrations. However, concentrations of up to 200 µM of compounds failed to inhibit PKG activity (assessed by phosphorylation of the established PKG substrates VASP, PDE5 and GRP2) in intact human platelets, rat mesangial cells and neonatal mouse cardiac myocytes. It should be noted that the measured PKG concentration is about 7 µM in platelets and 0.1 – 0.5 µM in all other tested cells. (D)-DT2 effects on platelet function did not correlate with PKG activity. Preincubation of platelets with 10 nM (D)-DT2 strongly inhibited thrombin-induced platelet aggregation and calcium mobilization, whereas it potentiated these effects in collagen-stimulated platelets.

## Conclusion

Interpretations of results based on PKG inhibitors require caution. None of the commercially available PKG inhibitors should be used without control experiments in intact cells since they may have unpredictable functional effects not mediated by PKG activity.
